# Places Maintained by Abortionists Are Nuisances

**Published:** 1907-06

**Authors:** 


### PLACES MAINTAINED BY ABORTIONISTS ARE NUISANCES.

One of the hardest problems to solve in the attempt to put professional abortionists out of business is the difficulty of obtaining direct evidence of a specific offense. Such evidence, of course, is essential if conviction is sought on the ground that the crime of aborption has been committed. If conviction can be secured on a more general charge, however, the problem seems in a fair way of being solved. nI our Medicolegal Department this week a decision of the Supreme Court of New York is reported which has a special significance in this regard, it being the first of its kind in American legal records. The defendant, an advertising abortionist, was charged with maintaining a public nuisance, which under the New York Penal Code is defined as: (1) Unlawfully doing an act or omitting to perform a duty, which act or omission annoys, injures or endangers the comfort of persons; or (2) offends public decency. The case was carried to the higher court after conviction of the defendant on the ground that the penal code made abortion a crime of a higher character and that there was no jurisdiction in the trial court of the offense charged as a public nuisance. No previous decision could be found declaring the maintenance of a place kept for such purposes a public nuisance. The court took the broad view that it was not necessary, as was technically claimed, that the actual commitment of the crime should be proved to bring the case under the statute. The offense of inviting the performance of abortion and maintaining premises for its commission is in itself a crime against order and public decency. A comprehensive definition such as was adopted by the court should cover a much larger class of offenses, it would seem, than those directly aimed at in the decision and it ought to be easy hereafter to put down illegal and immoral advertising offenders in New York. A valuable precedent is made also for legal proceedings against like offenses in other states where the technical interpretation of the letter and disregard of the spirit of the law have too often successfully aided open violators of good morals.—Ed. Jour. Amer. Med. Assy., May 11th, 1907.

